# Meal Microstructure Characterization from Sensor-Based Food Intake Detection

**DOI:** 10.3389/fnut.2017.00031

**Published:** 2017-07-17

**Authors:** Abul Doulah, Muhammad Farooq, Xin Yang, Jason Parton, Megan A. McCrory, Janine A. Higgins, Edward Sazonov

**Affiliations:** ^1^Department of Electrical and Computer Engineering, University of Alabama, Tuscaloosa, AL, United States; ^2^Department of Information Systems, Statistics, and Management Science, Culverhouse College of Commerce and Business Administration, University of Alabama, Tuscaloosa, AL, United States; ^3^Department of Health Sciences, Boston University, Boston, MA, United States; ^4^Department of Pediatrics, University of Colorado, Anschutz Medical Campus, Denver, CO, United States

**Keywords:** food intake detection, food diary, swallowing, chewing, wearable sensors, meal microstructure

## Abstract

To avoid the pitfalls of self-reported dietary intake, wearable sensors can be used. Many food ingestion sensors offer the ability to automatically detect food intake using time resolutions that range from 23 ms to 8 min. There is no defined standard time resolution to accurately measure ingestive behavior or a meal microstructure. This paper aims to estimate the time resolution needed to accurately represent the microstructure of meals such as duration of eating episode, the duration of actual ingestion, and number of eating events. Twelve participants wore the automatic ingestion monitor (AIM) and kept a standard diet diary to report their food intake in free-living conditions for 24 h. As a reference, participants were also asked to mark food intake with a push button sampled every 0.1 s. The duration of eating episodes, duration of ingestion, and number of eating events were computed from the food diary, AIM, and the push button resampled at different time resolutions (0.1–30s). ANOVA and multiple comparison tests showed that the duration of eating episodes estimated from the diary differed significantly from that estimated by the AIM and the push button (*p*-value <0.001). There were no significant differences in the number of eating events for push button resolutions of 0.1, 1, and 5 s, but there were significant differences in resolutions of 10–30s (*p*-value <0.05). The results suggest that the desired time resolution of sensor-based food intake detection should be ≤5 s to accurately detect meal microstructure. Furthermore, the AIM provides more accurate measurement of the eating episode duration than the diet diary.

## Introduction

An accurate understanding of dietary habits necessitates tracking of the dynamic process of each eating episode, known as meal microstructure (1–3). Meal microstructure includes factors such as eating episode duration (the duration from the start of the meal to the end including pauses), duration of actual ingestion (time spent eating in a given eating episode), the number of eating events (a bite, potentially followed by a sequence of chews and swallows), rate of ingestion, chewing frequency, chewing efficiency, and bite size (4). The meal microstructure is directly related to the ingestive behavior of individuals. Therefore, the study of meal microstructure may potentially yield new insights in the treatment of obesity and comorbid conditions. An accurate method of monitoring food intake is necessary to capture meal microstructure and provide a better understanding of eating behaviors. Further, such methods could potentially facilitate novel methods to reduce caloric intake and/or provide more effective self-assessment and feedback tools for those on a calorie restricted diet (5).

A wealth of preclinical data exists illustrating the importance of meal microstructure to caloric intake and weight control in animal models (6–8), but detailed human studies are rare. Among the few human studies that do exist, there are demonstrated differences in meal microstructure in obese versus lean people, men versus women, and among individuals in various states of health (9–11). Furthermore, larger food portion sizes have been shown to increase bite size and eating rate, leading to higher caloric intake in overweight women (12). In another study, eating the same meal slowly versus quickly increased energy expenditure *via* the thermic effect of food, and improved metabolic parameters (13). However, there are currently no free-living data from clinical studies which inform how these changes might affect caloric intake and weight control.

The scarcity of human data with regard to meal microstructure may be related to the difficulty of obtaining accurate data, especially in a community-dwelling situation, in which dietary intake and meal microstructure fluctuate more than in a controlled laboratory setting. Traditional methods of assessing dietary intake such as food frequency questionnaires, diet records, and 24-h dietary recall rely on self-report by participants (14–16). The self-reporting errors may be up to 50% of estimated intake, resulting in inaccurate assessment that may impact medical diagnosis or dietary interventions (17). The primary causes for inaccuracy in self-report include under or over reporting of all food items consumed and poor estimation of portion consumed. In addition, there is a possibility of a change in the eating behavior of individuals when they know they are being observed (18). Furthermore, traditional self-report methods do not provide important information about meal pattern/microstructure such as the number of bites or eating rate (19).

In an effort to monitor food intake behaviors, wearable sensor systems that integrate different sensor modalities have been proposed. Most of these methods use sensors that measure behavioral manifestations of eating, such as hand-to-mouth gestures, bites, chews, and swallows (20). Various approaches have been used, including an oral strain gauge sensor to measure tongue pressure and flexing during chewing (21), an ear-pad sound sensor to capture air-conducted vibrations while chewing (22), miniature microphones in the outer ear canal to capture chewing (23), an acoustic sensor worn around the neck to detect sounds made by the user’s mouth and throat while eating (24), a combination of a 3D gyroscope and 3 proximity sensors worn in an earpiece to measure ear canal deformations (25), a watch-like sensor (Bite Counter) to track wrist motion during hand-to-mouth gestures (26), a textile capacitive sensor worn as a neckband which detected swallowing and physical activity (27), a 3-axis accelerometer worn as a smartwatch to detect hand-to-mouth gestures (28), a piezoelectric sensor-based microphone to assess chewing and swallowing sounds (29) and others. Signal processing and pattern-recognition algorithms interpret the sensor signals to recognize food intake, often after segmenting the sensor signals into time intervals, or epochs, of fixed duration. The food intake recognition algorithm processes the fragment of the sensor signal within a given time interval and assigns it a label “food intake” or “not food intake.” Thus, the duration with which the sensor signal is segmented in turn determines the time resolution of food intake detection. Table 1 lists the studies mentioned earlier with respective time resolutions.

**Table 1:** Overview of food intake detection-related literature with sensor time resolution.

Reference	Sensor/device	Signal	Time resolution
Päßler et al. (23)	Miniature microphone	Body sound	23 ms
Amft et al. (22)	Acoustic sensor	Chewing sound	125 ms
Yatani and Truong (24)	Acoustic sensor	Body sound	186 ms
Dong et al. (26)	Inertial sensor	Arm movement	1 s
Rahman et al. (29)	Piezoelectric sensor	Chewing, swallowing	1–5 s
Sazonov et al. (31)	Acoustic sensor	Swallowing sounds	1.5 s
Farooq and Sazonov (35)	Piezoelectric strain sensor and accelerometer	Chewing and physical activity	3 s
Bedri et al. (25)	3D gyroscope, proximity	Ear canal deformations	5 s
Stellar and Shrager (21)	Oral strain gauge	Tongue pressure and flexing during chewing	Chart speed 5 s/in.
Thomaz et al. (28)	Inertial sensor	Arm movement	6 s
Sazonov and Fontana (32)	Piezoelectric strain gauge	Chewing	15, 30, and 60 s
Sazonov et al. (30)	Sensor	Chewing and swallowing frequency	30 s
Fontana et al. (33)	Piezoelectric strain gauge	Jaw motion	30 s
Farooq et al. (34)	Electroglottograph	Swallow	30 s
Cheng et al. (27)	Textile capacitive sensor	Swallow, swallow frequency, and physical activity	1.5–8 min

Our group has been developing systems to characterize food intake behavior by non-invasive monitoring of swallowing and chewing (30–33). In an earlier study (30), we proposed detection of food intake based on chewing and swallowing frequency. The instantaneous swallowing frequency was averaged over a sliding window of 30 s. In another study, the automatic detection of food intake was based on swallowing sounds using a high fidelity microphone placed over the laryngopharynx (31). The sound data were segmented into a series of overlapping epochs (375 ms, 750 ms, 1.5 s, and 3 s) with the 1.5 s epoch demonstrating the highest recognition accuracy. We then explored swallowing detection with an electroglottograph using epochs 30 s in duration (34). We have also proposed non-invasive monitoring of chewing using a piezoelectric strain gauge sensor (32). Three different time resolutions (15, 30, and 60 s) were evaluated to determine an appropriate window size for the detection of food intake with the 30 s resolution being most accurate. Recently, we presented and validated a novel wearable sensor system [automatic ingestion monitor (AIM)] for detecting food intake in community-dwelling conditions (33) by monitoring jaw motion. In that study, we used 30 s windows to recognize food intake. More recently, we modified the AIM to monitor the deformation of the temporalis muscle during food intake (35). In that study, the food intake was recognized with 99.85% accuracy and a time resolution of 3 s. Thus, we have established that the AIM detects food intake with a high degree of accuracy, but we have not yet determined which time resolution will provide the greatest accuracy in estimating the meal microstructure.

The purpose of this study was twofold: (1) to characterize meal microstructure, including duration of eating episodes, duration of actual ingestion, and number of eating events in community-dwelling healthy young adults using the AIM, and (2) to determine the optimal time resolution for doing so. The recommendations on time resolution may be used in future studies to accurately evaluate the practical capabilities of existing and emerging methods of food intake detection.

## Materials and Methods

### Data Collection

Experimental data were collected from a total of 12 participants (6 males and 6 females) with mean age 26.7 years (SD ± 3.7) and mean body mass index 24.4 kg/m^2^ (SD ±3.8). No individuals reported any medical condition that would affect normal food intake. All participants read and signed an informed consent document before the start of the experiment. The study was approved by the Internal Review Board at The University of Alabama. Each participant was asked to wear the sensor system (AIM) for a 24-h period where food intake was *ad libitum*. During the experiment, participants were able to perform daily living activities without restrictions.

The AIM consisted of data collection module, worn on a lanyard around the neck, and had interface for three different sensors:

(a) *Jaw motion sensor*—to detect characteristic motion of the jaw during chewing (32, 36). This sensor was attached directly below the ear using medical adhesive.
(b) *Hand gesture sensor*—to detect hand-to-mouth gestures associated with bites. It consisted of a radio-frequency transmitter worn on the inner side of the dominant arm and an RF receiver on the data collection module operating in radio-frequency identification band of 125 kHz.
(c) *Tri-axial accelerometer*—to detect body acceleration. This sensor was located in the data collection module.
(d) *Push button*—as the reference method for reporting food intake. The accuracy of push button report was tested in a controlled lab study (37) against video observation. The participants were asked to hold the push button in the non-dominant hand.

Sensor signals were acquired by the data collection module at a 1 kHz sampling frequency. All sensor signals were quantized with 12-bit resolution and transmitted *via* onboard Bluetooth to an Android smart phone. Apart from wearing the AIM, participants were also asked to keep a paper food diary noting the start and end times of each eating episode, what foods and beverages were consumed.

Participants were instructed to press the push button at the start of every bite of solid or semi-solid food or start of a chewing episode. The button was held down for the duration of chewing and released at the end of the chewing episode. The button was also used to report beverage intake by pressing and holding the button for the duration of the beverage ingestion episode.

### Food Intake Detection

Three different methods were used in this study to monitor food intake and meal microstructure: the food diaries, AIM, and push button. In the diary-based method, the food intake information was directly obtained from the completed food diaries. To detect the food intake using the AIM, a feature extraction algorithm and classification model developed in Ref. (33) was used. The model automatically recognized food intake with 30 s of resolution from AIM sensor signals.

### Meal Microstructure Analysis

**Figure 1:**
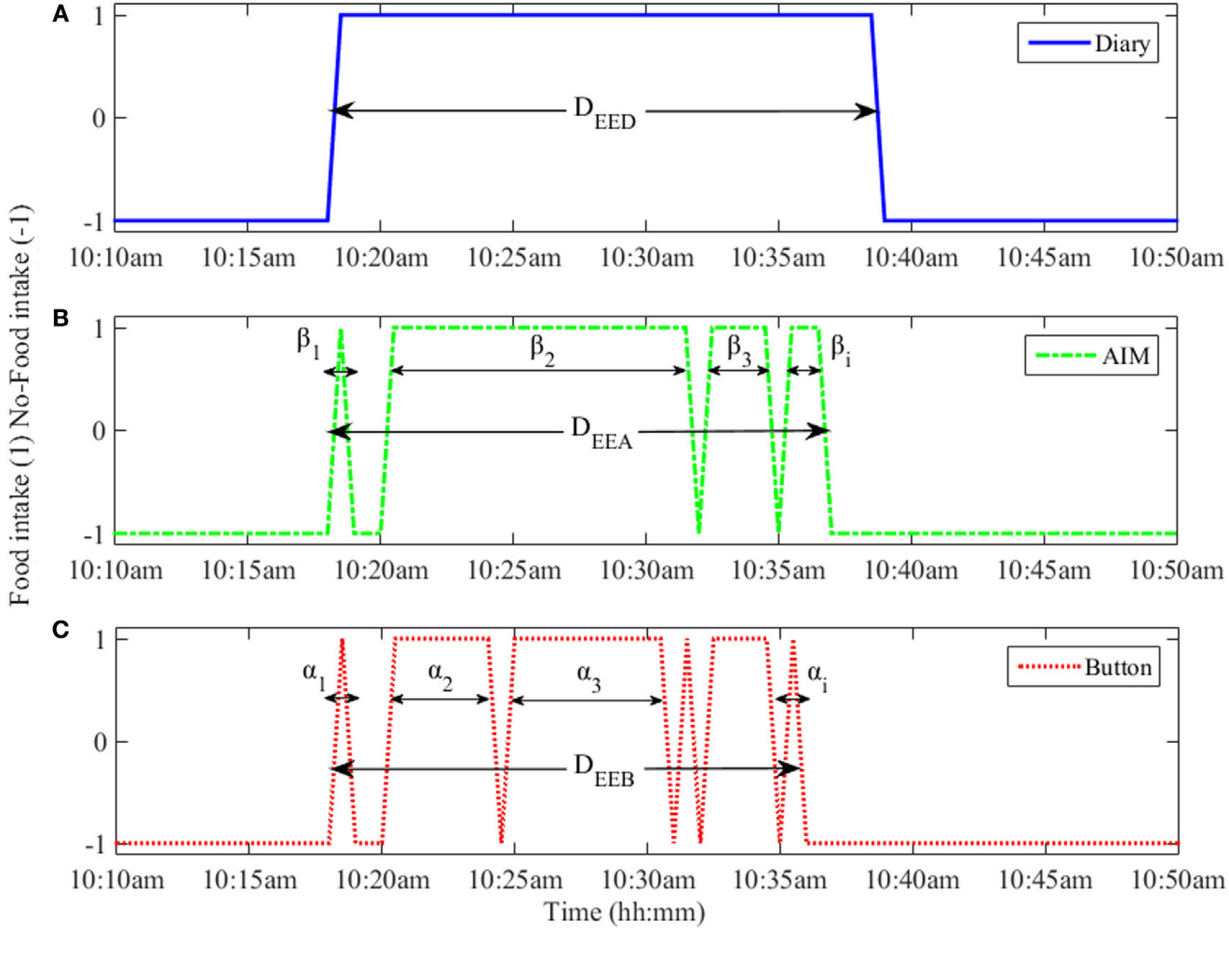
An eating episode reported by **(A)** the food diary, **(B)** automatic ingestion monitor (AIM), and **(C)** push button. The α*_i_* and β*_i_* represent the duration of individual eating event for push button and AIM, respectively. The *D*_EED_, *D*_EEA_, and *D*_EEB_ represent the eating episode duration from food diary, AIM, and push button, respectively.

The microstructure parameters of each eating episode were assessed from the food intake indicated by the methods described in Section “Food Intake Detection.” An example of a single eating episode assessed by each of these methods is shown in Figure 1.

The microstructure parameters extracted from the diary, AIM, and push button were:

*Number of eating events* (*N*), defined as the number of active ingestion segments in an eating episode (meal). The number of eating events for food diary (*N*_D_) was always 1. The number of eating events for button and AIM were represented as *N*_B_ and *N*_A_, respectively.
*Eating episode duration* (*D*_EE_), the duration between the start and stop times of eating episode, including segments without food intake. In the case of the food diary, the difference between the reported start and stop times was defined as the eating episode duration (*D*_EED_). The computation of duration from the AIM and the push button (*D*_EEA_ and *D*_EEB_) used the algorithm described below.
*Duration of actual ingestion* (*D*_I_), the duration of actual eating (subtracting any non-eating segments) within an eating episode. In the case of the food diary, the duration of actual ingestion (*D*_ID_) was the same as eating episode duration (*D*_EED_). The duration of each *i-*th atomic eating event for push button and AIM were expressed as α*_i_* and β*_i_* as shown in Figure 1 and durations of actual ingestion were computed as, $D_{IB}={\text{Σ}}_{i=1}^{N_{B}}\alpha_{i}$ and $D_{IA}={\text{Σ}}_{i=1}^{N_{A}}\beta_{i}.$

**Figure 2:**
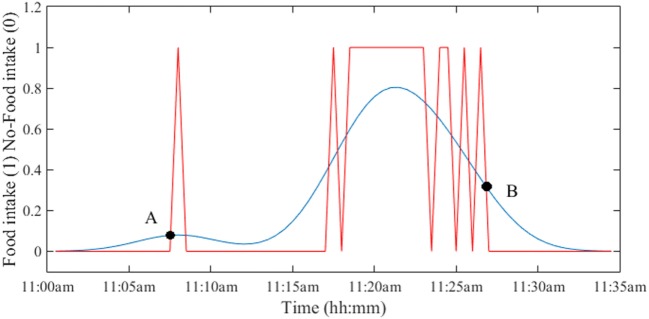
Determination of the duration of eating episode by using a Gaussian kernel function. The intersection of the original and smoothed signals provides the start time (point A) and end time (point B) of the eating episode. The duration of the eating episode is computed as the difference between time at points B and A.

The computation of *D*_EEA_ and *D*_EEB_ required determination of the boundaries of the eating episode. As shown in Figure 1, eating episodes may have had pauses and/or breaks within the meal that needed to be “smoothed” for the estimation of duration. To smooth the signal, a function called “kernel” is employed to compute the average of the neighboring data points. In this work, a smoothing kernel with the shape of a Gaussian (normal distribution, σ = SD) curve was used on the AIM and push button. The food intake detection by AIM was performed on 30 s intervals. To determine the suitable width of the kernel, the σ values from 1 to 6 (detection intervals) were tested. The optimal width was found to be at σ = 5 (150 s). Then, the start and end points of each eating episode were determined by the intersection of the original and Gaussian-smoothed signals as shown in Figure 2. The duration between A and B was defined as the eating episode duration. A script was written in MATLAB (Mathworks Inc., Natick, MA, USA) to compute all durations.

### Analysis of the Time Resolution

The signal from the push button reported food intake with the best time resolution (0.1 s) was used for the analysis of the optimal resolution to capture meal microstructure parameters. Different time resolutions can be used to best characterize different aspects of meal microstructure. For example, chewing events can be captured in short time resolution of 3 s. On the other hand, the detection of swallowing may need a window of as long as 30 s. We investigated a range of time resolutions in capturing the microstructure parameters. To test a range of time resolutions representative of methods in Table 1, the push button signal was resampled with progressively longer window sizes (1–30 s, representing the range of detection intervals reported in recent literature) using a resampling algorithm (38). The microstructure parameters were then computed from the resampled signal and tested for equality using the statistical analysis described below.

### Statistical Analysis

Statistical analysis was performed with SAS 9.0 (SAS Institute, Cary, NC, USA) and Matlab 2015 (Mathworks Inc., Natick, MA, USA). Differences in *D*_EE_ and *D*_I_ computed from the AIM, diary, and push button at time resolutions 0.1–30 s were analyzed with a linear mixed model with participant as a random factor. If the mixed model showed a significant difference among methods, Tukey–Kramer *post hoc* multiple comparisons analysis was performed to determine which methods differed from each other. Data for the *N*_B_ at different time resolutions were analyzed by one-way repeated ANOVA to determine whether different time resolution yielded differing results. Since the parametric method did not pass the residual diagnostic criteria, a non-parametric Friedman test method was adopted for repeated ANOVA. If the ANOVA results were significant, Tukey–Kramer *post hoc* multiple comparisons test was performed to determine which time resolutions differed from each other. To assess the relative bias (mean difference) and random error (1.96 SD of the difference) between methods, the Bland and Altman plots (39) were investigated. Statistical significance was assumed at *p*-value <0.05.

## Results

**Figure 3:**
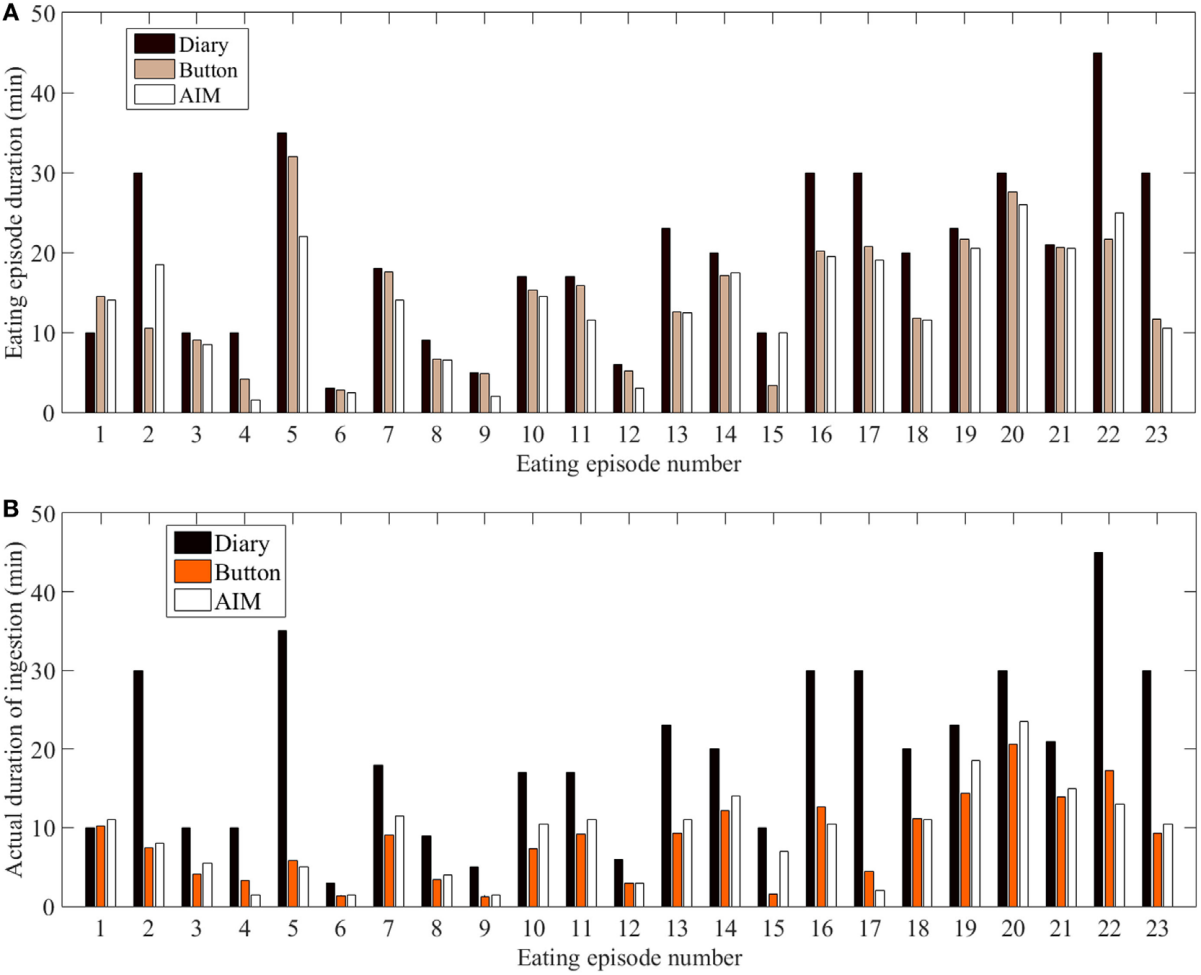
Duration across all eating episodes. **(A)** Eating episode duration. **(B)** Duration of actual ingestion.

**Figure 4:**
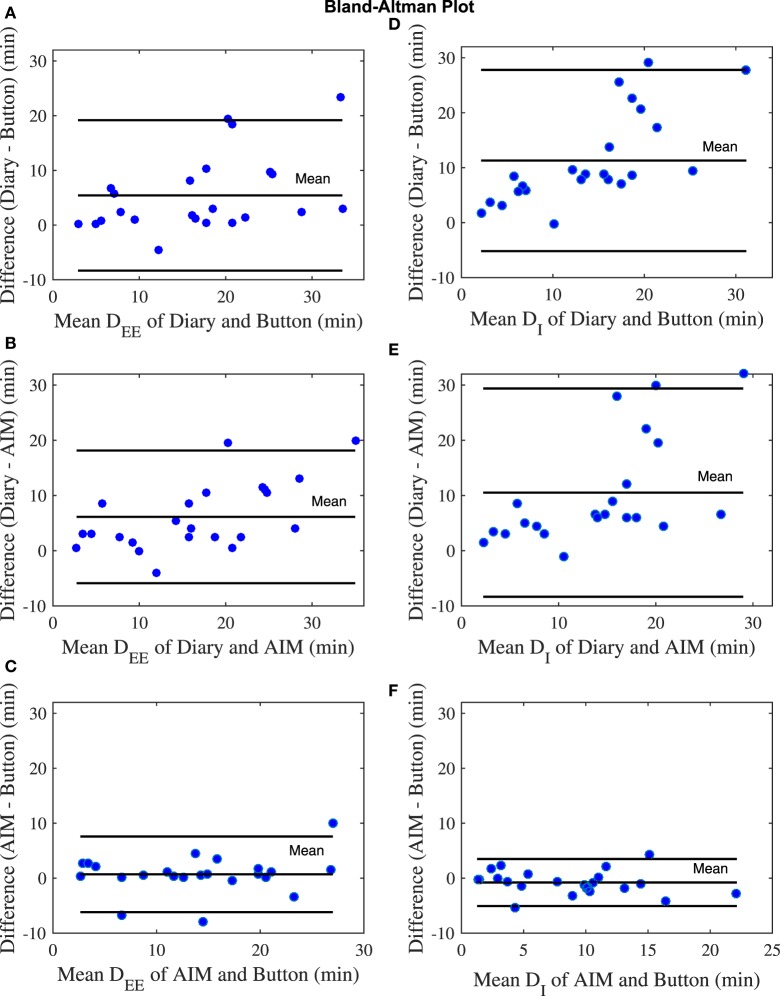
Bland–Altman plots for number of eating episodes. **(A)** The eating episode duration (*D*_EE_) of diary and button. **(B)** Duration of ingestion (*D*_I_) of diary and button. **(C)** *D*_EE_ of diary and automatic ingestion monitor (AIM). **(D)** *D*_I_ of diary and AIM. **(E)** *D*_EE_ of AIM and button. **(F)** *D*_I_ of AIM and button. The blue dots represent eating episodes.

In the study, out of the 12 participants, 4 participants did not provide time information for the start and end of the eating episodes in the food dairies. For the remaining 8 participants, 23 eating episodes had complete time information (based on the dairies), whereas 6 eating episodes had partial time information (e.g., only start time and no end time) and therefore only 23 eating episodes were included in the analysis. The mixed model showed that there were statistically significant differences in meal microstructure between the food diary, the AIM, and the push button. Multiple comparison analysis showed that the *D*_EE_ and *D*_I_ from the food diary differed significantly from the AIM (*p* < 0.001) and the push button (*p*-value <0.001), but the AIM and push button results did not differ from each other (Figure 3A). Participants’ self-reported meal durations from the food diary were significantly over-reported in comparison to the AIM and the push button (Figure 3B). The Bland–Altman analysis in Figure 4 shows good agreement between the AIM and push button methods but poor agreement between the diary and other methods. With regard to eating episode durations in Figures 4A–C, the limits of agreement between the AIM and the push button were narrower compared to the limits of agreement between the diary and the push button and the diary with the AIM. A similar narrow range of limit agreement was found for actual ingestion durations shown in Figure 4F.

**Figure 5:**
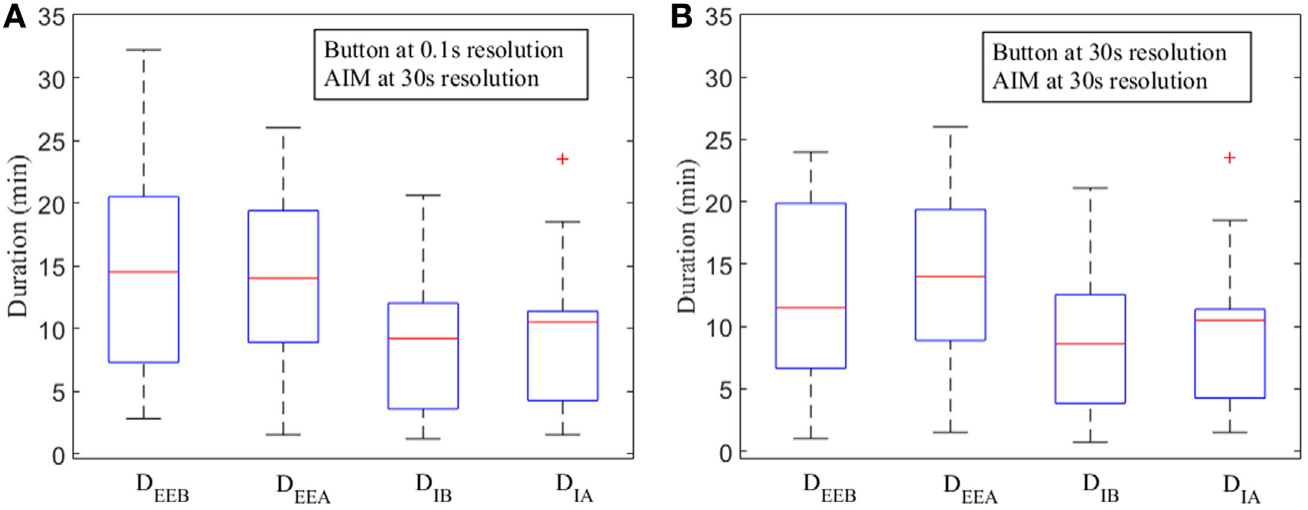
Box plots for measured durations of eating episodes [measured by push button—*D*_EEB_ and automatic ingestion monitor (AIM)—*D*_EEA_] and ingestion (button—*D*_IB_, AIM—*D*_IA_) at various time resolutions. **(A)** Push button at 0.1 s resolution and AIM at 30 s resolution. **(B)** Push button at 30 s and AIM at 30 s resolution. The red line indicates the median. Upper and lower whiskers show the minimum and maximum changes within 25th and 75th percentile, respectively. Lower and upper horizontal blue lines (on the box) indicate first and third quartile. Data points outside of the box are labeled as “outliers” and shown with a red cross.

The narrow degree of dispersions in the distribution of *D*_IB_ and *D*_IA_ compared to *D*_EEB_ and *D*_EEA_, respectively, indicate that the *D*_I_ is significantly smaller than the measured *D*_EE_ from boundaries (Figure 5).

**Figure 6:**
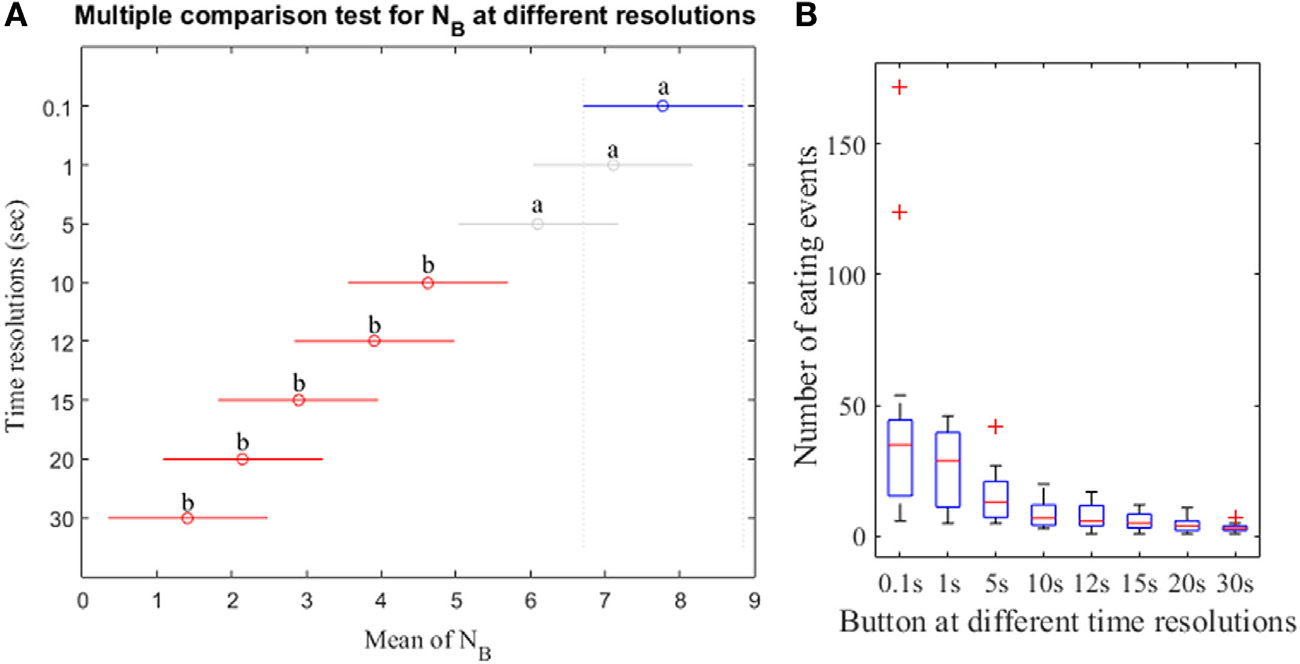
**(A)** *Post hoc* Tukey–Kramer test for the number of eating events from push button at different resolutions. The mean of *N*_B_ for each time resolution is represented by the symbol “o.” The letter “a” at the line indicates that the *N*_B_ is not significantly different whereas the letter “b” indicates the *N*_B_ is significantly different. **(B)** Distribution of *N*_B_ at different resolutions. Data points outside of the box are labeled as “outliers” and shown with a red cross.

Results of the non-parametric Friedman test shows that at least two *N* computed from different resolutions of the push button method were significantly different (*p*-value <0.05). *Post hoc* Tukey–Kramer test demonstrates that the *N*_B_ showed no significant differences for the high resolutions (0.1, 1, and 5 s) and exhibited differences for low resolutions (10–30 s; Figure 6A). The results also indicate that the low resolutions (e.g., 15 s) exhibit no significant differences for low resolutions (10–30 s) but significant differences for high resolutions (0.1, 1, and 5 s). Figure 6B demonstrates the box-plot distributions of *N*_B_ at different resolutions. For low resolutions, the *N*_B_ begins to exhibit small mean with compact distributions indicating the loss of meal microstructure information.

## Discussion

In this study, we compared the assessment of meal microstructure parameters between food diary, a wearable sensor (AIM) method, and a reference push button. In an effort to find a standard time resolution of food intake detection, we also provided an analysis of time resolution that may be used to accurately evaluate meal microstructure parameters. The major findings suggested that compared with the push button (reference method), the AIM sensor provided more accurate meal microstructure information relative to food diary. Furthermore, we found a sensor time resolution of 5 s was adequate to evaluate the meal microstructure parameters. These findings imply that to characterize meal microstructure, the AIM sensor-based dietary assessment method would be preferred over traditional food diary. Moreover, a sensor time resolution of <5 s should be used in future studies to best characterize meal microstructure using the sensor technologies.

Meal duration has significant effects on the total energy consumed (40), thus, accurate estimation of the meal/eating episode duration and duration of actual ingestion are very important for understanding eating behavior. We observed that the food diary over-estimated the eating occasion duration for most of the eating episodes compared with the AIM and reference method. One potential reason for the difference is the inherent reporting error of self-reported food diaries. On the other hand, the AIM does not depend on self-report; therefore, one would expect the AIM to be more accurate compared to the food diary. Bland–Altman analysis showed that the estimated durations from AIM had the lowest relative bias and the narrowest limit of agreement with the push button compared to food diary. These findings demonstrate that the AIM could provide more accurate information about the eating episode duration and actual ingestion duration in comparison to food diary. In addition, AIM could potentially offer information about the within meal behavior by examining meal microstructure. Therefore, the results suggest that unlike the food diary, the AIM can potentially detect the process of food ingestion and measure microstructure parameters without creating a reporting burden for the user. The AIM can potentially offer further characterization of the ingested foods analyzing the microstructure parameter that is not possible from food diary. An illustrative example is duration of actual ingestion versus duration of the eating episode. Even the best modern electronic diary is not capable of assessing the actual time spent eating and time spent in other activities during a meal. Use of sensor technology allows to measure these microstructure parameters and potentially use them as a metric in comparing food intake in different individuals.

Our findings suggest that the duration of actual ingestion was significantly lower than the eating episode duration because an eating episode typically consisted of several eating events and intra-meal pauses. These pauses can be short or long depending on individual eating habits or surroundings and could lead to substantial differences between eating episode duration and actual ingestion duration. The distributions of *D*_IB_, and *D*_IA_ were significantly compact and lower than the distributions of *D*_EEB_ and *D*_EEA_ (Figure 5). It is also evident that there was no significant difference between the median lines among respective time resolutions which implied that AIM could accurately estimate the duration of ingestion even with longer, 30 s resolution. To provide comparison with same resolution, push button signals was resampled, and durations were computed (Figure 5B). The *D*_EEB_ at 30 s resolution exhibited compact quartile ranges compared to *D*_EEB_ at 0.1 s. The median lines of both *D*_EEB_ and *D*_IA_ at 30 s started indicating small difference with the median lines of *D*_EEA_ and *D*_IA_ at 30 s. A potential reason could be the resampling of push button to 30 s that reduced the number of eating events with each eating episode.

The use of a range of time resolutions of sensor (1–30 s) was important because it represents the state of the art in sensor detection of food intake reported in recent literature. A part of reason is that the selection of time resolution for a given sensor is dependent on the physical phenomena being captured. For instance, the short duration may better capture the microstructure properties of food intake, but the time resolution may be limited by the nature of the physiological process used for detection of food intake. When using swallows to detect food intake, the ingestion is manifested as an increase in swallowing frequency from approximately 2 swallows per minute to >4 swallows per minute, thus limiting the time resolution to approximately 30 s (30). Chewing has a frequency of 0.94–2 Hz and therefore may employ a time resolution (otherwise known as detection window) as short as 3 s (35). Chewing sounds occur mostly in the range 1–2 kHz (41) and therefore, detection windows as short as 23 ms (23) were reported for the sounds. The study by Bellisle et al. (42) reported that consecutive bites are separated by 5–15 s for foods with different levels of palatability. In general, longer detection windows include more physiological events of interest (chews, swallows, etc.) and, therefore, may provide a higher accuracy compared to shorter detection windows. Thus, there is a potential tradeoff between optimal time resolution and the accuracy of food intake detection, which in turn has an impact on the accuracy of representing the meal microstructure. Results demonstrate that the time resolutions of 10–30 s indicated small *N*_B_ with compact distributions and resolutions of 0.1–5 s indicated comparatively spread distributions with large *N*_B_. Therefore, it can be inferred that the time resolutions of 0.1–5 s describe the meal microstructure accurately.

In the AIM sensor module, the jaw motion sensor signals were analyzed by means of chew detection to monitor food intake. The signals were divided into non-overlapping segments of 30 s due to the historical reasons of the technology development. However, the analysis of *N*_B_ suggested that the desired time resolution of sensor-based food intake detection should be ≤5 s to preserve the meal microstructure. Such window duration is potentially supported the frequency range of chewing (1.25–2.5 Hz) and use of shorter detection windows for detection of chewing by the AIM will be explored in the future.

The major strengths of this study were the performance comparison between food diary and AIM, and finding out a potential time resolution to capture meal microstructure by sensor-based methods. A limitation of this study was utilizing the push button as the reference method for assessing the microstructure of a meal. There is a possibility that using handheld push button might change the eating behavior of the participants as one hand is occupied by the button. While the button may have imposed some changes in hand use, many food are consumed with one hand only. This is especially true for quick ingestive events, such as grabbing and consuming a small food item. Therefore, we believe that the microstructure captured in this experiment is representative of real-world eating. The participants may also press or release the button accidentally and misreported the intake. While such errors are possible, hand button is arguably is one of the best ways to assess eating microstructure in free living. As our experience shows, the quality of video annotation of eating microstructure degrades greatly in conditions close to free living due to the difficulties in interpretation of complex ingestive behaviors (e.g., eating while talking). Great hand dexterity of humans (for example, demonstrated in complex button press combination in computer work and gaming) combined with self-perception (“feeling”) of the ingestion process allows for accurate representation even of transient events such as swallowing (37). As a potential alternative to handheld button, a foot pedal may potentially be utilized to record the ground truth so that the participant can use both hands while consuming the food. Further studies will need to involve more participants and a larger number of eating episodes. The future work should also investigate more complex microstructure parameters such as eating rate.

## Conclusion

Precise characterization of microstructural properties of a meal is a key to capturing of accurate eating patterns using sensor-based methods. Results suggested that the duration of the eating episodes estimated from food diaries was significantly different from the duration estimated by the AIM and push button and furthermore, that the AIM is more accurate that food diaries. Based on this work, the desired time resolution of sensor-based food intake detection should be ≤5 s to adequately document the meal microstructure parameters.

## Ethics Statement

This study was carried out in accordance with the recommendations of “the Internal Review Board at The University of Alabama” with written informed consent from all subjects. All subjects gave written informed consent in accordance with the Declaration of Helsinki. The protocol was approved by the “Internal Review Board at The University of Alabama.”

## Author Contributions

AD conceived of the statistical analysis for meal microstructure characterization, contributed to the analysis and interpretation of the data and drafting of the manuscript. MF contributed to the study design, coordination of the study, and designed algorithms for sensor-based food intake evaluation and contributed to the editing of the manuscript. XY contributed to the statistical analysis, interpretation of the data, and editing of the manuscript. JP, MM, JH, and ES contributed to interpretation of data and editing of the manuscript. ES conceived of the study and contributed to the study design, analysis, and interpretation of data and editing the manuscript. All authors have read and approved the final manuscript.

## Conflict of Interest Statement

The authors declare that the research was conducted in the absence of any commercial or financial relationships that could be construed as a potential conflict of interest.
